# Impact of the distance of spread through air spaces in non-small cell lung cancer

**DOI:** 10.1093/icvts/ivae181

**Published:** 2024-12-20

**Authors:** Asato Hashinokuchi, Takaki Akamine, Gouji Toyokawa, Kyoto Matsudo, Taichi Nagano, Fumihiko Kinoshita, Mikihiro Kohno, Takumi Tomonaga, Kenichi Kohashi, Mototsugu Shimokawa, Yoshinao Oda, Tomoyoshi Takenaka, Tomoharu Yoshizumi

**Affiliations:** Department of Surgery and Science, Graduate School of Medical Sciences, Kyushu University, Fukuoka, Japan; Department of Surgery and Science, Graduate School of Medical Sciences, Kyushu University, Fukuoka, Japan; Department of Thoracic Surgery, Clinical Research Institute, National Hospital Organization, Kyushu Medical Center, Chuo-ku, Fukuoka, Japan; Department of Surgery and Science, Graduate School of Medical Sciences, Kyushu University, Fukuoka, Japan; Department of Surgery and Science, Graduate School of Medical Sciences, Kyushu University, Fukuoka, Japan; Department of Surgery and Science, Graduate School of Medical Sciences, Kyushu University, Fukuoka, Japan; Department of Surgery and Science, Graduate School of Medical Sciences, Kyushu University, Fukuoka, Japan; Department of Pathology, Graduate School of Medical Sciences, Kyushu University, Fukuoka, Japan; Department of Pathology, Graduate School of Medicine, Osaka Metropolitan University, Osaka, Japan; Department of Biostatistics, Graduate School of Medicine, Yamaguchi University, Yamaguchi, Japan; Department of Pathology, Graduate School of Medical Sciences, Kyushu University, Fukuoka, Japan; Department of Surgery and Science, Graduate School of Medical Sciences, Kyushu University, Fukuoka, Japan; Department of Surgery and Science, Graduate School of Medical Sciences, Kyushu University, Fukuoka, Japan

**Keywords:** Non-small cell lung cancer, Spread through air spaces, Maximal spread distance

## Abstract

**OBJECTIVES:**

Spread through air spaces (STAS) is considered a poor prognostic factor in patients with resected non-small lung cell cancer; however, the clinical significance of STAS extent remains unclear. We hypothesized that the further the tumour cells spread from the tumour edge, the worse the prognosis becomes.

**METHODS:**

This study retrospectively reviewed the data of 642 patients with completely resected pathological stage I–III non-small lung cell cancer between 2008 and 2018. The maximum spread distance (MSD) from the tumour edge to the farthest STAS was quantitatively evaluated, and STAS was categorized as limited (MSD ≤1000 μm) or extended (MSD >1000 μm), based on the median MSD. Recurrence-free survival (RFS) and overall survival (OS) were compared among patients stratified by STAS classification.

**RESULTS:**

Patients were classified into STAS-negative (*n* = 382, 59.6%), limited STAS (*n* = 130, 20.2%) and extended STAS (*n* = 130, 20.2%) groups. Extended STAS was associated with a high maximum standardized uptake value, advanced pathological stage and vascular invasion compared with limited STAS. The extended STAS group demonstrated significantly shorter RFS and OS than both the limited STAS and STAS-negative groups (both *P* < 0.001 for RFS; *P* = 0.007 and *P* < 0.001 for OS, respectively). Multivariable analysis showed that extended STAS was an independent prognostic factor for both RFS and OS (*P* < 0.001, *P* < 0.001, respectively).

**CONCLUSIONS:**

The distance from tumour edge to STAS affects prognosis in patients with completely resected non-small lung cell cancer.

**Clinical registration number:**

IRB approval number: 2019-232

## Graphical Abstract

**Figure:**
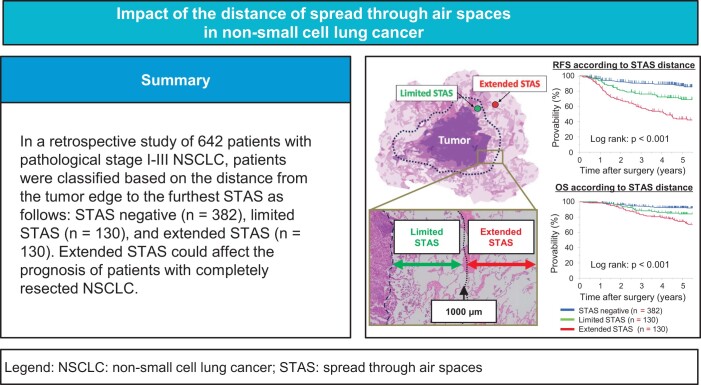

## INTRODUCTION

‘Spread through air spaces’ (STAS) in non-small cell lung cancer (NSCLC), introduced into the 2015 World Health Organization (WHO) classification, is defined as the spread of micropapillary cluster (MPC), solid nest and/or single cancer cells into air spaces in the lung parenchyma beyond the edge of the primary tumour [1]. Although STAS is considered a novel invasive pattern [2], its characterization is debated, particularly its potential to be an artefact caused by tissue manipulation during specimen handling and processing [3, 4]. Some have contended that loose tumour fragments in air spaces are merely artefacts, questioning the invasive implications of STAS [5]. However, numerous studies have demonstrated a strong association of STAS with lymphovascular invasion, high recurrence rate and poor prognosis in NSCLC [2, 6, 7]. Recently, the International Association for the Study of Lung Cancer Staging recommended that STAS should be included as a histological descriptor in the 9th Edition of the TNM classification of lung cancer [8]. Moreover, three-dimensional immunohistochemical analyses have shown that tumour clusters can adhere to distant alveolar walls, suggesting that it may be a true invasive process rather than an artefact [9]. Despite ongoing debate, the clinical significance of STAS in NSCLC is gaining increased recognition.

A possible reason for the controversy regarding STAS is the lack of objective assessment criteria. Several studies have quantitatively assessed STAS and observed a correlation between a higher quantity of STAS and worse prognostic outcomes [10]. Xie *et al.* [11] investigated the prognostic significance of morphological subtypes of STAS, identifying MPC and solid nest STAS, but not single-cell STAS, as independent prognostic factors. The prognostic impact of STAS extent, defined as the distance from the tumour edge to the STAS, remains controversial [12]. Given the established correlation between the anatomic extent of tumours and prognosis, we hypothesized that STAS distance plays an important role in the prognosis of patients with NSCLC. Herein, we investigated the associations among STAS distance, clinicopathological factors and the prognosis of patients with resected NSCLC.

## MATERIALS AND METHODS

### Study design and patients

We retrospectively identified 776 patients with lung cancer who underwent surgery between January 2008 and December 2018 at Kyushu University Hospital. Patients who underwent incomplete resection (*n* = 27) or neoadjuvant therapy (*n* = 42) and those diagnosed with neuroendocrine carcinoma (*n* = 38) or other rare histological types (*n* = 27) were excluded. Finally, 642 patients with completely resected pathological stages I–III NSCLC (lung adenocarcinoma and squamous cell carcinoma) were identified. The study flow diagram of eligible patients is shown in Supplementary Material, Fig. S1. All patients were restaged according to the 8th edition of the TNM classification [13]. Additional information on the surgical procedure, adjuvant chemotherapy and follow-up is available in Supplementary Method.

### Histopathological evaluation of ‘spread through air spaces’

STAS was defined as the presence of tumour cells within lung parenchymal air spaces extending beyond the edge of the tumour [14]. To distinguish genuine STAS from artefacts, we applied the following previously reported exclusion criteria: (i) mechanically dissociated tumour floaters, (ii) normal benign pneumocytes or bronchial cells, (iii) strips of tumour cells detached from alveolar walls or stroma and (iv) isolated tumour clusters distantly situated from the tumour rather than extending continuously from the tumour edge to the farthest point within adjacent air spaces [15].

**Figure 1:**
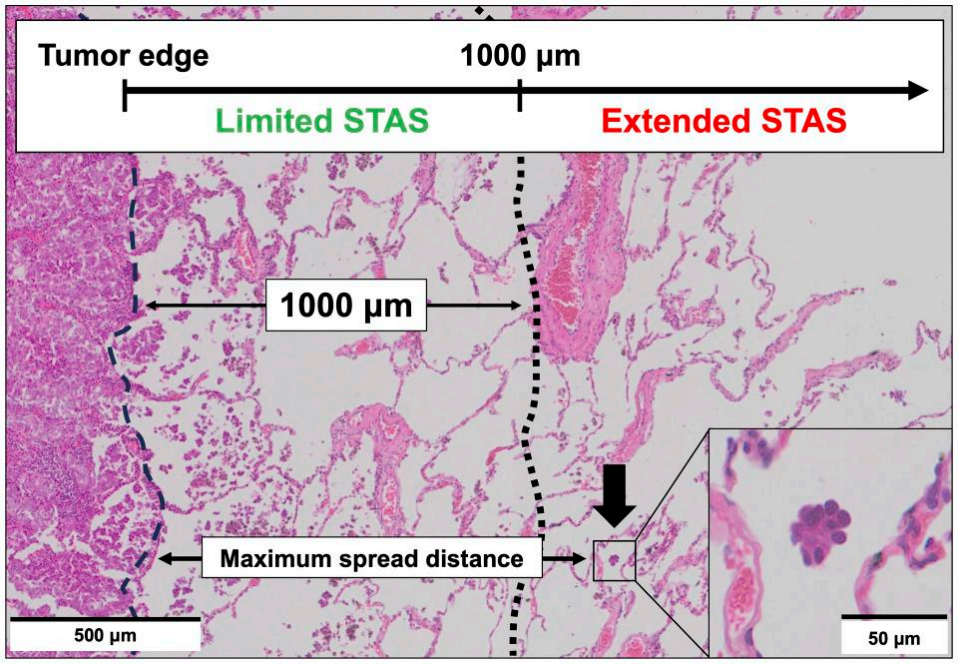
Measurement of maximum spread distance and definition of extended and limited spread through air spaces (STAS). An example of lung adenocarcinoma exhibiting extended STAS (scale bar: 500 μm) and the furthest STAS (arrow, scale bar: 50 μm).

STAS was classified into single cell, MPC and solid nest patterns (Supplementary Material, Fig. S2) [11]. Two investigators (A.H. and A.T.) and 1 pathologist (T.T. or K.K.) retrospectively assessed STAS and categorized it into 3 patterns. The maximum spread distance (MSD) between the tumour edge and the farthest STAS was measured using a ruler and was categorized into limited STAS (MSD ≤1000 μm) and extended STAS (MSD >1000 μm) based on the median MSD (Fig. 1). We recorded the number of STAS and classified them into low (1–4 clusters) and high STAS (≥5 clusters), as reported previously [10].

### Statistical analysis

The relationships between the clinicopathological factors and presence of STAS or the STAS distance were assessed using unpaired *t*-tests. Recurrence-free survival (RFS, the period from surgery to the date of recurrence, or any cause of death of patients without event being censored at the follow-up date) and overall survival (OS, the period from surgery to the date of death of patients without event being censored at the follow-up date) were calculated using the Kaplan–Meier method with log-rank tests. Univariable and multivariable analyses, using Cox proportional hazards regression, were performed to assess the relationships among RFS, OS and clinicopathological features. The backward elimination method was used for multivariable Cox proportional hazards regression, which initially included all variables, and the variable with the highest *P*-value was iteratively removed until *P*-value <0.05. Multivariable Cox regression of STAS characteristics for RFS and OS was adjusted for age, sex, smoking status, histological type, surgical procedure, pathological stage and pleural, lymphatic and vascular invasion. A *P*-value <0.05 indicated statistical significance. All statistical analyses were performed using JMP (version 16.0).

## RESULTS

### Clinicopathological characteristics

Supplementary Material, Table S1 shows the clinicopathological characteristics of 642 patients with completely resected NSCLC. The median patient age was 69 years (range, 23–89 years); 337 (62.2%) patients were male and 283 (44.1%) were smokers. Herein, 452 (70.4%), 77 (12.0%), 63 (9.8%) and 50 (7.8%) patients had pathological stages IA, IB, II and III, respectively. STAS was identified in 260 of the 642 patients (40.5%).

### Characteristics of patients with ‘spread through air spaces’

The median MSD for STAS was 1000 μm, which was adopted as the threshold for subsequent analyses. Accordingly, STAS-positive cases were categorized into 2 groups: extended (*n* = 130, MSD >1000 μm) and limited STAS (*n* = 130, MSD ≤1000 μm). STAS morphological patterns included single cells [*n* = 54 (20.8%)], MPC [*n* = 145 (55.8%)] and solid nests [*n* = 61 (23.4%)]. Based on the number of STAS, STAS-positive patients were classified as low [63 patients (24.2%)] or high STAS [197 patients (75.8%)].

We investigated the relationship between MSD and STAS morphological patterns. The median MSDs in patients with single cell, MPC and solid nest STAS were 300, 1200 and 1000 μm, respectively. MSDs were significantly longer in patients with MPC and solid nest STAS than in those with single-cell STAS (*P* < 0.001); however, no significant difference in MSD between MPC and solid nest STAS (*P* = 0.365) was observed.

### Clinicopathological factors based on ‘spread through air spaces’ distance

Table 1 shows clinicopathological factors according to the presence of STAS and STAS distance. The presence of STAS was significantly associated with sex (*P* = 0.008), smoking status (*P* = 0.028), surgical procedure (*P* < 0.001), histological status (*P* = 0.029), maximum standardized uptake value (*P* < 0.001), pathological stage (*P* < 0.001), lymphatic invasion (*P* < 0.001) and vascular invasion (*P* = 0.002). Furthermore, extended STAS was markedly associated with maximum standardized uptake value (*P* = 0.009), pathological T (pT) status (*P* = 0.027) and vascular invasion (*P* = 0.008) compared with limited STAS.

**Table 1:** Association between the presence of STAS and clinicopathological factors in all patients, and between maximum spread distance and clinicopathological factors in STAS-positive patients

Factors	All patients (*n* = 642)					STAS-positive patients (*n* = 260)				
	STAS-negative (*n* = 382)		STAS-positive (*n* = 260)		*P*-value	Limited STAS (*n* = 130)		Extended STAS (*n* = 130)		*P*-value
	*n* (%)		*n* (%)			*n* (%)		*n* (%)		
Age										
≥70 years	185	(48.4)	130	(50.0)	0.696	65	(50.0)	65	(50.0)	1.00
Sex										
Male	184	(48.2)	153	(59.9)	0.008	73	(56.2)	80	(61.5)	0.378
Smoking										
Smoker	200	(52.4)	159	(61.2)	0.028	75	(57.7)	84	(64.6)	0.252
Surgical procedure										
Lobectomies	238	(62.3)	211	(81.2)	<0.001	106	(81.5)	105	(80.8)	0.874
Histological subtype										
Adenocarcinoma	341	(89.3)	245	(94.2)	0.029	120	(92.3)	125	(96.2)	0.184
SUV_max_^a^										
≥3.0	133	(39.6)	151	(65.1)	<0.001	66	(56.9)	85	(73.3)	0.009
Pathological stage										
II–III	46	(12.0)	68	(26.2)	<0.001	28	(21.5)	40	(30.8)	0.090
Pleural invasion^a^										
Present	57	(15.0)	49	(18.9)	0.186	20	(15.5)	29	(22.3)	0.162
Lymphatic invasion										
Present	15	(3.9)	41	(15.8)	<0.001	15	(11.5)	26	(20.0)	0.061
Vascular invasion										
Present	66	(17.3)	71	(27.3)	0.002	26	(20.0)	45	(34.6)	0.008

a Only available data were considered for analysis.

STAS: spread through air space; SUV_max_: maximum standardized uptake value.

### Survival analysis according to ‘spread through air spaces’ distance

**Figure 2:**
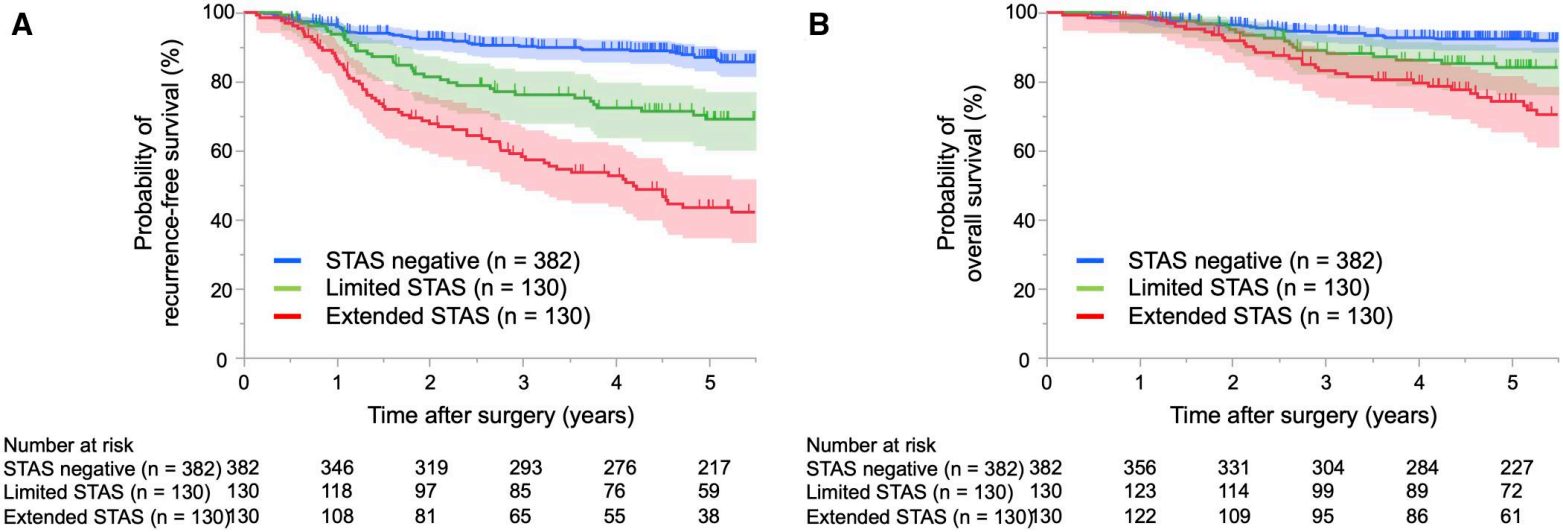
Kaplan–Meier curves of recurrence-free survival (**A**) and overall survival (**B**) in patients with pathological stages I–III resected non-small cell lung carcinoma (NSCLC) according to the maximum distance of spread through air spaces (STAS).

The numbers of recurrences and any cause of deaths were 142 (22.2%) and 96 (15.0%), respectively. Figure 2 presents RFS and OS curves based on STAS distance. Patients with extended STAS exhibited significantly worse RFS than STAS-negative patients and those with limited STAS (*P* < 0.001 and *P* < 0.001, respectively; Fig. 2A). Similarly, the OS was significantly shorter for patients with extended STAS than for those who were STAS-negative or had limited STAS (*P* < 0.001 or *P* = 0.007, respectively; Fig. 2B). Furthermore, patients with limited STAS had worse RFS and OS than STAS-negative patients (*P* < 0.001 and *P* = 0.022). In the additional analysis in pathological stage I, extended STAS was related to significantly shorter RFS than limited STAS and STAS-negative (*P* = 0.017 and *P* < 0.001, respectively), although there was no significant difference between extended and limited STAS (*P* = 0.133) (Supplementary Material, Fig. S3).

Multivariable analysis showed that limited and extended STAS were independent prognostic factors for RFS [hazard ratio (HR): 2.23; 95% confidence interval (CI): 1.47–3.39; *P* < 0.001 and HR: 3.56; 95% CI: 2.42–5.23; *P* < 0.001, respectively; Table 2]. Limited and extended STAS were also an independent prognostic factor for OS (HR: 1.81; 95% CI: 1.04–3.16; *P* = 0.037 and HR: 3.28; 95% CI: 2.03–5.30; *P* < 0.001, respectively; Supplementary Material, Table S2).

**Table 2:** Univariable and multivariable analyses of recurrence-free survival in patients with completely resected NSCLC

Factors		Univariable analysis		Multivariable analysis	
		HR (95% CI)	*P*	HR (95% CI)	*P*
Age	≥70 years/<70 years	1.73 (1.27–2.36)	<0.001	1.96 (1.43–2.68)	<0.001
Sex	Male/female	1.72 (1.25–2.36)	<0.001		
Smoking	Smoker/non-smoker	1.69 (1.22–2.33)	0.002	1.52 (1.08–2.13)	0.017
Histological type	Squamous/adenocarcinoma	1.97 (1.23–3.15)	0.005	2.75 (1.67–4.54	<0.001
Surgical procedure	Sublobar resection/lobectomies	1.50 (1.04–2.16)	0.060		
Pathological T	T2–4/T1	3.31 (2.43–4.50)	<0.001	1.70 (1.15–2.52)	0.007
Pathological N	N1–2/N0	5.26 (3.79–7.30)	<0.001	2.70 (1.80–4.05)	<0.001
Pleural invasion	Present/absent	3.78 (2.73–5.23)	<0.001	1.55 (1.02–2.34)	0.039
Lymphatic invasion	Present/absent	4.82 (3.34–6.97)	<0.001	1.63 (1.04–2.55)	0.034
Vascular invasion	Present/absent	3.61 (2.64–4.93)	<0.001	1.56 (1.07–2.26)	0.021
STAS (distance)	Negative	1		1	
	Limited	2.38 (1.58–3.58)	<0.001	2.23 (1.47–3.39)	<0.001
	Extended	4.95 (3.47–7.07)	<0.001	3.56 (2.42–5.23)	<0.001

CI: confidence interval; HR: hazard ratio; NSCLC: non-small cell lung cancer; STAS: spread through the air spaces.

### Additional survival analysis according to ‘spread through air spaces’ morphological subtypes

**Figure 3:**
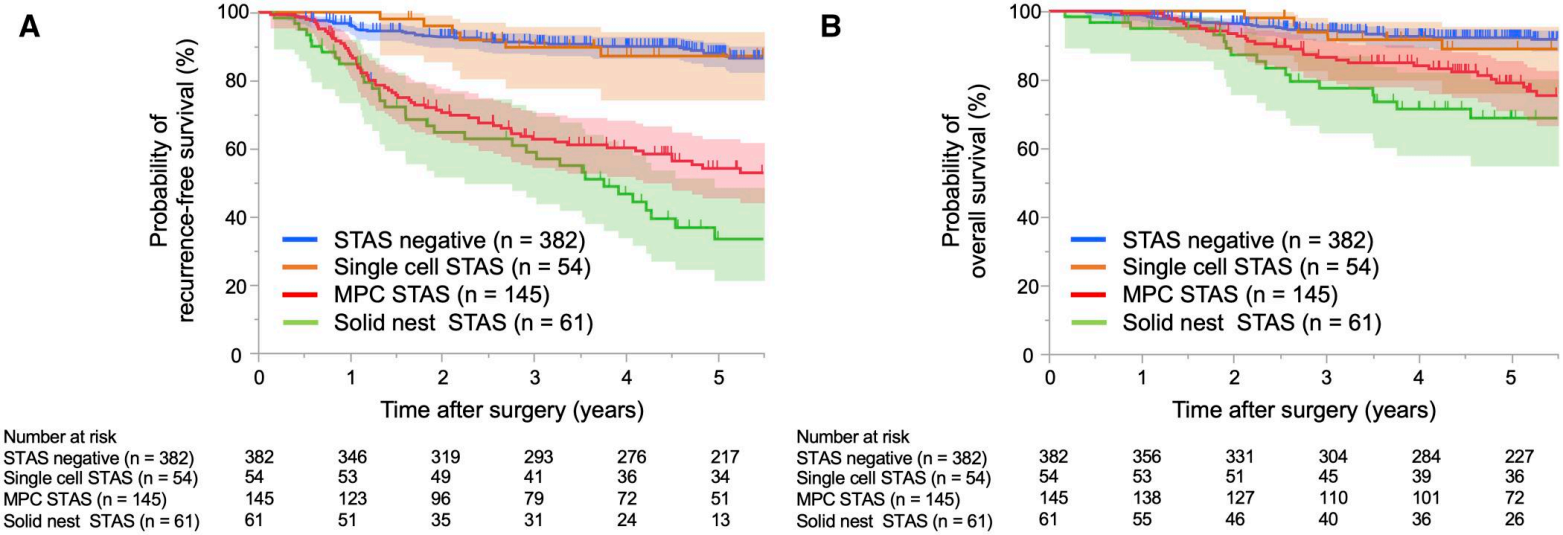
Kaplan–Meier curves of recurrence-free survival (**A**) and overall survival (**B**) in patients with pathological stages I–III resected non-small cell lung carcinoma (NSCLC) according to the morphological subtypes of spread through air spaces (STAS): single cell, micropapillary cluster (MPC) and solid nest patterns.

Regarding STAS morphological subtypes, patients with MPC or solid nest STAS had significantly worse RFS than those with single-cell STAS (*P* < 0.001 and *P* < 0.001, respectively) and STAS-negative patients (*P* < 0.001 and *P* < 0.001, respectively) (Fig. 3A). Additionally, patients with MPC or solid nest STAS had significantly worse OS than those with single-cell STAS (*P* < 0.001 and *P* < 0.001, respectively) and STAS-negative patients (*P* < 0.001 and *P* < 0.001, respectively) (Fig. 3B). Notably, no prognostic differences in RFS or OS were observed between patients with single-cell STAS and STAS-negative patients (*P* = 0.99 and *P* = 0.70, respectively), as shown in Fig. 3.

### Survival analysis stratified by the classification of spread through air spaces characteristics

To elucidate factors contributing to the prognosis based on STAS, we performed a multivariable Cox regression analysis for RFS and OS (Supplementary Material, Table S3). When adjusted for clinicopathological factors, extended STAS was significantly associated with poor RFS and OS compared with limited STAS (adjusted HR: 1.84, 95% CI: 1.23–2.75, *P* = 0.003 and adjusted HR: 1.95, 95% CI: 1.15–3.33, *P* = 0.014, respectively). High STAS was not significantly related to RFS or OS compared with low STAS. MPC and solid nest STAS were significantly associated with poor RFS, but not with OS, compared with single-cell STAS (adjusted HR 3.93, 95% CI: 1.90–8.14, *P* < 0.001).

### Impact of extended spread through air spaces on the survival of pathological T1 non-small cell lung cancer

In patients with pathological T1 NSCLC, extended STAS was associated with significantly poorer RFS and OS than STAS-negative and limited STAS (*P* < 0.001 and *P* = 0.024 for RFS; *P* < 0.001 and *P* = 0.047 for OS, respectively; Supplementary Material, Fig. S4). No significant differences in RFS and OS were observed for extended STAS in pathological T1 and T2 (*P* = 0.61 and *P* = 0.70, respectively), as shown in Supplementary Material, Fig. S4.

## DISCUSSION

Herein, we investigated the prognostic significance of the distance from the tumour edge to the furthest STAS in patients with resected NSCLC. The T factor in the TNM classification measures the local extent of the tumour. Therefore, we hypothesized that further extension of tumour cells from the tumour edge leads to a worse prognosis. Based on the MSD of STAS, we classified patients into STAS-negative, limited STAS and extended STAS groups. Patients with extended STAS exhibited higher maximum standardized uptake value, advanced pathological stage and more malignant pathological features than those with limited STAS. Multivariable analysis revealed that extended STAS was an independent prognostic factor for both RFS and OS.

Six prior studies reported the prognostic significance of STAS distance (Supplementary Material, Table S4) [2, 6, 16–19]. However, the methodologies used to define STAS distance varied among these studies. Warth *et al.* [2] and Lu *et al.* [6] defined STAS distance using the number of alveoli between the furthest STAS and the tumour edge and observed no significant prognostic impact of STAS distance in NSCLC. Conversely, Han *et al.* [17] defined STAS distance as the distance from the tumour edge to the farthest STAS, which is consistent with our study, and demonstrated that extended STAS, defined as ≥2500 μm distance of the furthest STAS from the tumour edge, was an independent poor prognostic factor in NSCLC regardless of tumour stage. They also showed that extended STAS correlated with advanced stage and pathological invasive features compared with limited STAS. Taken together, these results indicate that the clinical significance of the STAS distance may depend on how the cut-off value is determined, and further studies are warranted to establish a method for measuring the STAS distance and define the clinically relevant cut-off value.

A biological perspective may help to explain the differences in the prognosis among extended STAS, limited STAS and STAS-negative patients. STAS was correlated with lower E-cadherin and higher vimentin expression in NSCLC, indicating that STAS could be associated with the epithelial–mesenchymal transition, a biological process that promotes tumour cell migration and invasion [20]. However, Yagi *et al.* [9] reported that the surviving tumour cells of STAS exhibited positive cytoplasmic staining for E-cadherin, similar to that observed in the primary tumour. Thus, it remains unclear whether STAS tumour cells actually undergo epithelial–mesenchymal transition. Notably, the biological significance of varying STAS distances is yet to be explored. Future studies should aim to elucidate the biological mechanisms of STAS to deepen our understanding of STAS in NSCLC.

Given that the T factor is assigned based on the anatomical extent of the primary tumour, we hypothesized that pT1 NSCLC with extended STAS corresponds to a higher T stage. Our study indicated that the prognosis of patients diagnosed with pT1 lung NSCLC with extended STAS was similar to that of patients diagnosed with pT2 NSCLC. This result aligns with that of a previous study showing that the prognosis of STAS-positive patients diagnosed with stage IA cancer (tumours >2–3 cm) was similar to that of patients diagnosed with stage IB cancer [21]. Recently, the presence of STAS was suggested to be useful in the staging of lung cancer as well as pleural invasion [8]. These findings suggest that STAS may significantly influence the determination of pathological T status in the future.

However, an objective assessment method for STAS has not been established. The positivity rate for STAS varies among previously reported studies, ranging from 15.0% to 51.4% [22]. STAS has been comprehensively evaluated based on the distribution, morphology and the number and distance of tumour cells; however, the minimum number of tumour cells or the necessary distance from the tumour edge has not been established [22]. Therefore, establishing the criteria for STAS assessment according to its objective pathological characteristics, such as morphological subtypes, number and distance, is essential. Herein, no significant prognostic differences were observed between single-cell STAS and STAS-negative patients; thus, single-cell STAS may be a mechanical artefact, which is consistent with a previous study [11]. Therein, the number of STAS was not significant predictor of RFS and OS in the multivariable Cox regression analysis. In previous study, the number of artefact STAS seemed equivalent to that of genuine STAS [11]. However, STAS distance was significantly associated with RFS and OS, and STAS distance with single-cell STAS was significantly shorter than other subtypes in this study (300 μm vs 1200 μm), suggesting that evaluating STAS distance could aid in distinguishing genuine STAS from artefact STAS. Furthermore, the method for evaluating STAS distance is simple and objective, and therefore, STAS distance could improve the assessment of STAS.

The STAS distance may be useful for making treatment decisions in patients with NSCLC. Adjuvant chemotherapy is the standard treatment for patients with stages II and III NSCLC after complete resection; however, recent studies indicated that adjuvant chemotherapy can benefit even in stage I lung adenocarcinoma patients with STAS [23, 24]. Lv *et al.* [23] showed that adjuvant chemotherapy improved the prognosis of stage IB STAS-positive patients with high-risk recurrence factors, such as poor differentiation, lymphovascular invasion and pleural invasion. Our study demonstrated that extended STAS is a risk factor of poor prognosis in STAS-positive NSCLC; therefore, adjuvant chemotherapy can contribute to better prognosis of stage I patients with extended STAS. Further studies are warranted.

### Limitation

The strength of our study is the detailed classification of STAS. However, this study had several limitations. First, this was a single-centre retrospective study. Second, the STAS distance could have been influenced by formalin fixation. Even in the same tumour, the MSD may be not identical in the different sections of the tumour. Indeed, the MSD in our study tended to be slightly shorter than that in previous studies (6.18). Therefore, large-scale, multicentre prospective studies investigating the optimal cut-off values to define extended STAS and their validity are warranted.

## CONCLUSION

Our findings show that the distance from the tumour edge to the furthest STAS can affect the prognosis of patients with completely resected NSCLC. Therefore, this study highlights the importance of the presence and grading of STAS according to the MSD in pathological assessment.

## Supplementary Material

ivae181_Supplementary_Data

## Data Availability

The data underlying this article will be shared on reasonable request to the corresponding author.

## ABBREVIATIONS

CI: Confidence interval
HR: Hazard ratio
MPC: Micropapillary cluster
MSD: Maximum spread distance
NSCLC: Non-small cell lung cancer
OS: Overall survival
pT: Pathological T
RFS: Recurrence-free survival
STAS: Spread through air spaces
WHO: World Health Organization
