# Supplementary material for: Impact of the distance of spread through air spaces in non-small cell lung cancer
Source: Interdiscip Cardiovasc Thorac Surg. 2024 Dec 20;40(1):ivae181. doi: 10.1093/icvts/ivae181 (PMC11669314; doi:10.1093/icvts/ivae181)
Supplement: ivae181_Supplementary_Data [file ivae181_supplementary_data.zip › CORRECT Supplemental Method.docx]

*Ethical statement*

Each patient provided written informed consent for the use of their medical records. This study was approved by the Institutional Review Board of Kyushu University Hospital (approval number: 2019-232).

*Surgical procedure and adjuvant chemotherapy*

The procedures and approach of the operation were decided on the basis of surgeon preference, considering factors such as tumor size, location, patient comorbidities, and perceived surgical risks. Lymph node evaluation was not routinely performed in wedge resection.

In Japan, postoperative adjuvant chemotherapy using uracil–tegafur is recommended for patients with completely resected pathological stage I NSCLC with a total tumor size >2 cm. In addition, for patients with completely resected pathological stage II-III NSCLC, platinum-based chemotherapy was given. However, the decision to administer the adjuvant therapy was determined by the surgeon, after taking into account the patient’s age and comorbidities.

*Follow-up*

Following surgical resection, routine checkups (including physical examination, blood tests, and chest radiography) were performed at 3-month intervals for the first 3 years and at 6-month intervals thereafter. Computed tomography (CT) was performed twice each year for the first 3 years and at 1-year intervals thereafter. If recurrent disease was suspected, further evaluations, such as head magnetic resonance imaging and 2-[18F]-fluoro-2-deoxy-D-glucose positron emission tomography, were performed. Recurrent disease was diagnosed based on physical examinations and diagnostic imaging findings. When clinically feasible, diagnoses were confirmed histologically. The date of recurrence was defined based on either a histologically proven recurrence or when a recurrent disease was recognized by the attending physician in cases diagnosed based on clinical evidence.
