# Supplementary material for: Impact of the distance of spread through air spaces in non-small cell lung cancer
Source: Interdiscip Cardiovasc Thorac Surg. 2024 Dec 20;40(1):ivae181. doi: 10.1093/icvts/ivae181 (PMC11669314; doi:10.1093/icvts/ivae181)
Supplement: ivae181_Supplementary_Data [file ivae181_supplementary_data.zip › CORRECT Supplemental Table.docx]

**Supplemental Table 1**. Clinicopathological characteristics of patients with NSCLC who underwent surgery (n = 642)

| **Characteristic** | **n (%)** | |
| --- | --- | --- |
| Age (years; range) | 69 (23–89) | |
| Sex | Male | 337 (62.2%) |
|  | Female | 305 (37.8%) |
| Smoking status | Non-smoker | 359 (55.9%) |
|  | Smoker | 283 (44.1%) |
| Surgical procedure | Lobectomy | 449 (69.9%) |
|  | Sublobar resection | 193 (30.1%) |
| Histological type | Adenocarcinoma | 586 (91.3%) |
|  | Squamous cell carcinoma | 56 (8.7%) |
| Pathological T | T1 | 488 (76.0%) |
|  | T2 | 121 (18.8%) |
|  | T3 | 22 (3.5%) |
|  | T4 | 11 (1.7%) |
| Pathological N | N0 | 560 (87.2%) |
|  | N1 | 40 (6.2%) |
|  | N2 | 42 (6.6%) |
| Pathological stage | IA | 451 (70.2%) |
|  | IB | 77 (12.0%) |
|  | II | 65 (10.1%) |
|  | III | 49 (7.7%) |
| Pleural invasion^a^ | Negative | 533 (83.0%) |
|  | Positive | 106 (17.0%) |
| Lymphatic invasion | Negative | 586 (91.3%) |
|  | Positive | 56 (8.7%) |
| Vascular invasion | Negative | 505 (78.7%) |
|  | positive | 137 (21.3%) |
| STAS | STAS negative | 382 (59.5%) |
|  | STAS positive | 260 (40.5%) |

a: Cases for which data were available.

NSCLC, non-small cell lung cancer; STAS, spread through air spaces.

**Supplemental Table 2**. Univariable and multivariable analyses of overall survival in patients with completely resected NSCLC

| **Factors** |  | **Univariable Analysis** | | **Multivariable Analysis** | |
| --- | --- | --- | --- | --- | --- |
|  |  | **HR (95% CI)** | ***P*** | **HR (95% CI)** | ***P*** |
| Age | ≥70 years/<70 years | 2.40 (1.58–3.64) | <.001 | 2.79 (1.82–4.28) | <.001 |
| Sex | male/female | 1.64 (1.08–2.48) | .019 |  |  |
| Smoking | smoker/non-smoker | 1.35 (0.89–2.03) | .158 |  |  |
| Histological type | squamous/adenocarcinoma | 2.35 (1.31–4.22) | .004 | 3.51 (1.91–6.45) | <.001 |
| Surgical procedure | sublobar resection/lobectomies | 1.58 (0.96–2.58) | .070 |  |  |
| Pathological T | T2-4/T1 | 3.10 (2.07–4.64) | <.001 | 2.20 (1.82–4.28) | <.001 |
| Pathological N | N1-2/N0 | 3.21 (2.05-5.01) | <.001 |  |  |
| pleural invasion | present/absent | 2.51 (1.60–3.92) | <.001 |  |  |
| lymphatic invasion | present/absent | 3.86 (2.39–6.23) | <.001 | 2.07 (1.22–3.51) | .007 |
| vascular invasion | present/absent | 3.14 (2.09–4.72) | <.001 | 1.95 (1.22–3.11) | .005 |
| STAS | Negative | 1 | | 1 | |
|  | limited | 1.86 (1.08–3.21) | .025 | 1.81 (1.04–3.16) | .037 |
|  | extended | 3.78 (2.40–5.95) | <.001 | 3.28 (2.03–5.30) | <.001 |

CI, confidence interval; NSCLC, non-small cell lung carcinoma; HR, hazard ratio; STAS, spread through air spaces.

**Supplemental Table 3**. Cox multivariable analysis of the classification of STAS characteristics for RFS and OS in patients with completely resected NSCLC

| **Factors** | **Adjusted HR for RFS (95% CI)** | ***P* value** | **Adjusted HR for OS (95% CI)** | ***P* value** |
| --- | --- | --- | --- | --- |
| Maximum spread of distance |  |  |  |  |
| extended vs. negative | 3.91 (2.68–5.71) | <.001 | 3.06 (1.88–4.96) | <.001 |
| limited vs. negative | 2.12 (1.40–3.21) | <.001 | 1.56 (0.90–2.73) | .116 |
| extended vs. limited | 1.84 (1.23–2.75) | .003 | 1.95 (1.15–3.33) | .014 |
| Number |  |  |  |  |
| high vs. negative | 3.00 (2.09–4.29) | <.001 | 2.48 (1.57–3.92) | <.001 |
| low vs. negative | 2.25 (1.35–3.74) | .002 | 2.42 (1.26–4.63) | .008 |
| high vs. low | 1.33 (0.82–2.17) | .245 | 1.03 (0.55–1.93) | .936 |
| Morphology |  |  |  |  |
| MPC and solid nest vs. negative | 3.64 (2.57–5.15) | <.001 | 2.78 (1.78–4.33) | <.001 |
| single cell vs. negative | 0.93 (0.44–1.95) | .840 | 1.28 (0.53–3.05) | .584 |
| MPC and solid nest vs. single cell | 3.93 (1.90–8.14) | <.001 | 2.18 (0.93–5.09) | .072 |

CI, confidence interval; HR, hazard ratio; MPC, micropapillary cluster; NSCLC, non-small cell lung cancer; OS, overall survival; RFS, recurrence-free survival; STAS, spread through air spaces.

**Supplemental Table 4.** Summary of previous reports focusing on STAS distance

| **Author (references)** | **Histological**  **type** | **Median of MSD** | **Cut-off value of MSD** | **No. of patients for analysis** | **No. of STAS-positive patients** | | **Outcome in prognosis** |
| --- | --- | --- | --- | --- | --- | --- | --- |
|  |  |  |  |  | **Limited STAS** | **Extended STAS** |  |
| Warth et al. | Ad | NA | 3 alveolae | 569 | 123 | 165 | Not significant |
| Lu S et al. | SCC | 1400 μm  (400–3200 μm) | 3 alveolae | 445 | 32 | 100 | Not significant |
| Yanagawa et al. | SCC | 800 μm  (300–2800 μm) | 800μm | 220 | 26 | 16 | Not significant |
| Khalil et al. | Ad | 2200 μm | Not determined | 787 | NA | | Not significant |
| Stogbauer | SCC | NA | 181 μm | 203 | 150 | 53 | Poor prognosis in OS |
| Han et al. | Ad | NA | 2500 μm | 1544 | 393 | 291 | Poor prognosis in  both DFS and OS |
| Present study | NSCLC | 1000 μm  (100–7400 μm) | 1000 μm | 642 | 130 | 130 | Poor prognosis in  both RFS and OS |

Ad, adenocarcinoma; DFS, disease-free survival; MSD, maximum spread distance; NA, not available; NSCLC, non-small cell lung carcinoma; OS, overall survival; RFS, recurrence-free survival; SCC, squamous cell carcinoma; STAS, spread through air spaces.
