# Supplementary material for: Impact of the distance of spread through air spaces in non-small cell lung cancer
Source: Interdiscip Cardiovasc Thorac Surg. 2024 Dec 20;40(1):ivae181. doi: 10.1093/icvts/ivae181 (PMC11669314; doi:10.1093/icvts/ivae181)
Supplement: ivae181_Supplementary_Data [file ivae181_supplementary_data.zip › CORRECT Supplemental Figure Legends.docx]

**Supplemental Figure Legends**

**Supplemental Figure 1.**

Summary of study cohorts.

**Supplemental Figure 2.**

Morphological subtypes of STAS include single cell (A), MPC (B), and solid nest patterns (C). Tumor cells in the single cell pattern are detected (arrows). Scale bar: 100 μm. MPC, micropapillary cluster; STAS, spread through air spaces.

**Supplemental Figure 3.**

Kaplan–Meier curves of recurrence-free survival (A) and overall survival (B) in patients with pathological stage I completely resected non-small cell lung carcinoma (NSCLC) according to the maximum spread distance of spread through air spaces (STAS).

**Supplemental Figure 4.**

Kaplan–Meier curves of recurrence-free survival (A) and overall survival (B) in patients with pathological T1-2 resected NSCLC stratified by the maximum spread distance of STAS. NSCLC, non-small cell lung cancer; STAS, spread through air spaces.
